# *Clostridioides difficile* ribotype distribution in a large teaching hospital in Serbia

**DOI:** 10.1186/s13099-020-00364-7

**Published:** 2020-05-22

**Authors:** Miloš Korać, Maja Rupnik, Nataša Nikolić, Milica Jovanović, Tanja Tošić, Jovan Malinić, Nikola Mitrović, Marko Marković, Ankica Vujović, Sanja Peruničić, Ksenija Bojović, Vladimir Djordjević, Aleksandra Barać, Ivana Milošević

**Affiliations:** 1 University of Belgrade, Faculty of Medicine, Dr Subotića 8, 11000 Belgrade, Serbia; 2 University Hospital for Infectious and Tropical Diseases, Clinical Centre of Serbia, Bulevar oslobođenja 16, 11000 Belgrade, Serbia; 3 Microbiology Department, Clinical Centre of Serbia, Pasterova 4, Belgrade, Serbia; 4 Department for Microbiological Research, Centre for Medical Microbiology, National Laboratory for Health, Environment and Food, Prvomajska 1, 2000 Maribor, Slovenia; 5 University of Maribor, Faculty of Medicine, Taborska 8, 2000 Maribor, Slovenia; 6 Clinic for Digestive Surgery, Clinical Centre of Serbia, Dr Koste Todorovića 6, 11000 Belgrade, Serbia

**Keywords:** *Clostridium difficile*, Ribotype 027, Serbia

## Abstract

**Background:**

The global epidemic of nosocomial diarrhea caused by *Clostridioides* (*Clostridium) difficile* started in 2000, with high mortality rates and emergence of a new hypervirulent strain NAP1/BI/027. The aim of this study was to assess the presence of ribotype 027 and other *C. difficile* ribotypes in a Serbian University Hospital, compare the temporal variability of ribotypes 3 years apart, as well as to compare clinical, demographic and laboratory characteristics and disease outcome among patients infected with 027 and non-027 ribotype. This was a prospective observational cohort study addressing 4-month intervals during 2014/2015 and 2017/2018.

**Results:**

Ribotyping was performed in 64 non-duplicate *C. difficile* strains. Ribotype 027 was the most prevalent, and was detected in 53 (82.8%) patients (43/45 and 10/19 patients in 2014–2015 and 2017/2018, respectively). Other detected ribotypes were 001/072 in 4 (6.3%), 002 in 4 (6.3%), 014/020 in 2 (3.1%) and 176 in 1 (1.5%) patient. The percentage of the patients infected with ribotype 027 significantly decreased during the 3-year period, from 95.6 to 52.6% (*p *< 0.001). Ribotype 027 infection was associated with fluoroquinolone treatment more frequently than infection with other ribotypes [33 (62.3%) vs. 2 (18.2%), *p *= 0.010)]. A severe *C. difficile* infection was diagnosed more often in patients with the detected ribotype 027 compared to those infected with non-027 ribotypes (p = 0.006). No significant difference in the mortality and recurrence rates was found between the patients infected with ribotype 027 and those infected with other ribotypes [10/53 (18.8%) vs. 2/11 (18.2%), p = 0.708, and 10/35 (28.6%) vs. 0/2 (0%), p = 1.000, respectively].

**Conclusion:**

*Clostridium difficile* ribotype 027 was the most prevalent ribotype among patients in a large Serbian hospital, but there is a clear decreasing trend.

## Highlights

1. Ribotype 027 was most prevalent among the 64 detected *C. difficile* strains.
2. Ribotype 027 infection was associated with fluoroquinolone treatment more frequently than infection with other ribotypes.
3. A severe *C. difficile* infection was diagnosed more often in patients with ribotype 027 compared to those infected with non-027 ribotypes.
4. Ribotype 027 infection did not influence mortality and recurrence rates compared to infection with other ribotypes.
5. The number of patients infected with ribotype 027 significantly decreased during the 3-year period.

## Background

The global epidemic of nosocomial diarrhea caused by *Clostridioides (Clostridium) difficile* started in 2000 in the United States and Canada [1]. Increased prevalence and higher mortality rates were registered in 2001, especially among the elderly. NAP1/BI/027 (North American pulsed-field 1/PCR-ribotype 027), a new hypervirulent strain associated with severe *C. difficile* infection (CDI) was discovered in 2002 [1]. The majority of the NAP1/BI/027 isolates are resistant to fluoroquinolones. They also possess virulence factors such as an increased sporulation and modified surface layer protein adherence [2]. The NAP1/BI/027 strain has been detected in almost all European countries, with a different prevalence rate.

The reports on *C. difficile* in Serbia are scarce [3–11]. Since 2008, the University Hospital for Infectious and Tropical Diseases of the Clinical Center of Serbia in Belgrade has been the central Serbian institution for the treatment of patients with CDI [3]. Among 510 patients treated in this hospital from 2008 to 2013, the reported recurrence and case fatality rates were 8.4% and 6.3%, respectively [3]. More severe forms of CDI and a notably higher recurrence rate were observed among patients treated in the same hospital from 2013 to 2015 [6]. This suggested a potential outbreak of the ribotype 027 and emphasized the need for an analysis of the distribution of the *C. difficile* ribotypes among patients in Serbia. Furthermore, it was important to evaluate whether the subsequent implementation of the rational antibiotics usage influenced the incidence of different ribotypes in the following period. To the best of our knowledge, there have been no published reports on the *C. difficile* ribotypes in Serbia addressing the period after 2015.

The aim of this study was to assess the presence of the ribotype 027 and other *C. difficile* ribotypes in a large university hospital in Serbia, compare the temporal variability of ribotypes 3 years apart, as well as to compare clinical, demographic and laboratory characteristics and disease outcome among patients infected with ribotype 027 and non-027.

## Methods

### Study setting and patient population

The study was carried out in the University Hospital for Infectious and Tropical Diseases of the Clinical Centre of Serbia in Belgrade, which is a tertiary-care facility where patients with infectious diseases from the entire country are referred. The study was designed as a prospective, observational cohort study addressing two intervals, 3 years apart. The first interval was from November 1 2014 to February 28 2015 and the second from November 1 2017 to February 28 2018. The patients with confirmed CDI treated during these intervals as inpatients in the abovementioned hospital were eligible for inclusion in the study. The severity of the disease was defined by the IDSA/SHEA (Infectious Disease Society of America/Society of Hospital Epidemiologists of America) criteria [1].

### *Clostridium difficile* diagnosis and strains characterization

In patients with diarrhea, microbiological tests and/or endoscopic finding of pseudomembranous colitis, followed by histological analyses of biopsy samples established the diagnosis of CDI. Microbiological testing was based on the detection of glutamat dehydrogenase (GDH) (RIDA^®^QUICK *Clostridium difficile* GDH, R-Biopharm AG, Darmstadt, Germany) and in the case of a positive GDH test a rapid test for detection of *C. difficile* toxin A and B in stool *(*RIDA^®^QUICK *Clostridium difficile* Toxin A/B,R-Biopharm AG, Darmstadt, Germany) was performed. When available, stool culture for *C. difficile* (medium Chrom ID *C. difficile,* bioMérieux, 69280, Marcy l’Etoile, France) was also performed using GDH and toxin positive stools.

The ribotyping of the *C. difficile* strains was performed in the National Laboratory for Health, Environment and Food, Centre for Medical Microbiology, Department for Microbiological Research in Maribor, Slovenia. *C. difficile* strains were typed with agarose based ribotyping with Bidet primers as described by Janezic and Rupnik [12].

Due to limited possibilities for strains typing, two 4-month intervals, 3 years apart, were designated for collecting the samples for the study.

However, for various reasons, the number of isolated and characterized strains presented in this study is lower than the number of patients admitted for CDI during the defined intervals. Some patients’ stool cultures were negative for *C. difficile*, although they had positive GDH and toxin A/B tests. In some patients, the diagnosis of CDI was established based on typical endoscopic findings, while microbiological criteria were not fulfilled. Furthermore, the medium for stool culture was not always available. In addition, some of the stored *C. difficile* strains could not be recultivated for typing.

### Statistical analyses

Demographic characteristics (age, gender), clinical presentation and severity of the disease (fever, abdominal cramps, vomiting, abdominal distension, hypotension, initial number of stools, presence of loose (mucoid) stool and ascites), baseline laboratory analyses (complete blood count, C-reactive protein (CRP), albumin concentration) and disease outcome (resolution of diarrhea, death or recurrence) were compared among patients infected with ribotype 027 and other *C. difficile* ribotypes. All statistical analyses were performed using an electronic database organized in SPSS version 21.0. The independent-samples t test was used to compare the means. The non-parametric variables were analyzed using the Chi-square or Fisher’s exact test, as appropriate. The level of significance was 0.05.

Ethical approval for the study was obtained from the Clinical Center of Serbia Ethics Committee.

All procedures performed involving human participants were in accordance with the ethical standards of the institutional research committee and with the 1964 Helsinki declaration and its later amendments or comparable ethical standards. Informed consent was obtained from all individual participants (or their caregivers) included in the study.

## Results

Fifty-seven *C. difficile* strains were cultivated from CDI patients admitted to hospital from November 1, 2014 to February 28, 2015 and 19 *C. difficile* strains from the samples taken during the second study period (November 1 2017–February 28 2018). Out of these 76 strains, only 64 (45 from the first and 19 from the second study period) were typed since there was no growth in 12 samples during recultivation.

Ribotype 027 was the most prevalent among 64 typed strains and was found in 53 (82.8%) patients. Other isolated *C. difficile* ribotypes were 001/072, 002, 014/020 and 176 which were detected in 4 (6.3%), 4 (6.3%), 2 (3.1%) and 1 (1.5%) patient, respectively. The percentage of patients infected with ribotype 027 significantly decreased during the 3-year period, from 95.6% (43/45) in 2014–2015 to 52.6% (10/19) in 2017–2018 (*p *< 0.001) (Table 1).

**Table 1:** Clinical and laboratory characteristics of patients with CDI infected with ribotype 027 and other ribotypes

	Ribotype 027 (N = 53) n (%)	Other ribotypes (N = 11) n (%)	*p*
2014/2015 (N = 45)	43 (95.6%)	2 (4.4%)	< 0.001
2017/2018 (N = 19)	10 (52.6%)	9 (47.4%)	
Age > 65 years	45 (84.9%)	8 (72.7%)	0.330
Fever	23 (43.3%)	3 (27.3%)	0.076
Abdominal cramps	44 (83%)	5 (45.5%)	0.007
Vomiting	9 (16.9%)	0	0.140
Abdominal distension	7 (13.2%)	1 (9%)	0.805
Hypotension	23 (43.4%)	6 (54.5%)	0.499
Initial number of stools (n)	1.67 ± 0.8	1.50 ± 0.7	0.766
Loose (mucoid) stools	44 (83%)	7 (63.4%)	0.325
CRP > 100 mg/l	21 (39.6%)	5 (45.5%)	0.353
WBC > 15 × 10^9^/l	27 (50.9%)	1 (9%)	0.274
Albumin < 30 g/l	34 (64.1%)	7 (63.6%)	0.618
Ascites	7 (13.2%)	1 (9%)	0.707
Time to resolution of diarrhea (days)	5.5 ± 2.8	6.8 ± 4.8	0.275
Severe CDI	41 (77.3%)	4 (36.4%)	0.006
Severe, complicated CDI	5 (9.4%)	1 (0.9%)	1.000
First episode	29 (54.7%)	4 (36.4%)	0.637

*WBC* white blood cell count, *CRP-C* reactive protein

All study patients received antibiotics for various reasons during the period of 2 months preceding CDI. The majority of patients, 35/64 (54.7%), were previously treated with fluoroquinolones, while 23/64 (35.9%) and 6/64 (9.4%) patients were treated with cephalosporins and carbapenems, respectively. Patients infected with ribotype 027 received fluoroquinolones prior to CDI more often than patients infected with other ribotypes [33/53 (62.3%) vs. 2/11 (18.2%), *p *= 0.010)]. The rate of the patients receiving fluoroquinolones decreased significantly over time [29/45 (64.5%) during the first vs. 6/19 (31.6%) during the second interval, *p *= 0.027]. These and other data about previous antibiotic usage according to different ribotypes and study periods are shown in Table 2.

**Table 2:** Antibiotic usage prior to *C. difficile* infection according to different ribotypes and study intervals

Previously used antibiotics	Ribotype 027 N = 53	Other ribotypes N = 11	*p*	2014/2015 N = 45	2017/2018 N = 19	*p*
Fluoroquinolones n (%)	33 (62.3%)	2 (18.2%)	0.010	29 (64.5%)	6 (31.6%)	0.027
Cephalosporins n (%)	18 (33.9%)	5 (45.4%)	0.504	15 (33.3%)	8 (42.1%)	0.573
Carbapenems n (%)	2 (3.8%)	4 (36.4%)	0.006	1 (2.2%)	5 (26.3%)	0.007

Clinical and laboratory characteristics of patients infected with 027 and non-027 ribotypes were compared (Table 1). The mean leukocyte count in the patients with ribotype 027 infection was significantly higher in comparison to the patients infected with other ribotypes (16.31 ± 8.4 × 10^9^/l vs.10.39 ± 4.14 × 10^9^/l, *p *= 0.027). The patients infected with ribotype 027 manifested more often a severe form of the disease (*p *= 0.006), but no significant difference was found in the mortality and recurrence rates between the patients infected with 027 and non 027 ribotypes [10/53 (18.8%) vs. 2/11 (18.2%), p = 0.708, and 10/35 (28.6%) vs. 0/2 (0%), p = 1.000, respectively].

Two (16%) patients suffering from 027 ribotype infection died of severe, complicated disease, and the remaining ten (84%), regardless of the ribotype, due to their underlying comorbidities. Risk factors for fatal outcome in patients infected with ribotype 027 were high leucocyte count (22.74 ± 9.75 × 10^9^/l in those who died vs. 14.7 ± 7.07 × 10^9^/l, in those who survived, *p *= 0.003), high CRP (168.86 ± 61.63 mg/l vs. 97.07 ± 73.96 mg/l, p = 0.006) and creatinine level (209.25 ± 170.03 mg/l vs. 123.06 ± 71.13 mg/l, p = 0.017).

## Discussion

The University hospital for Infectious and Tropical Diseases of the Clinical Center of Serbia in Belgrade is the biggest infectious diseases facility in Serbia. Over 2700 patients with CDI were treated in this institution from 2008 to 2018. The overall number of CDI patients treated annually decreased notably from 494 patients treated in 2015, to 391 patients in 2016, 322 patients in 2017, and 275 patients in 2018 (unpublished data).

Despite the huge number of treated patients, only 64 non-duplicate strains were typed and analyzed in the present study for the reasons stated above.

The vast majority of patients in the study had been infected with the hypervirulent NAP1/BI/027 strain. During the period of 2014/2015, the rate of the patients infected with this ribotype was as high as 95.6%, suggesting that NAP1/BI/027 was the causative agent of the CDI outbreak in Serbia. The high rate of the ribotype 027 infection was also observed in Romania (82.6%) and Poland (82.4%) during 2013/2014 [13, 14]. The study results reflecting the predominance of NAP1/BI/027 infection are in accordance with the previously reported data on the distribution of the *C. difficile* ribotypes in southeastern European countries [4]. According to Rupnik et al., 027 ribotype was isolated in 65.8% of samples from Serbia and neighboring Bosnia and Herzegovina [4]. The other most prevalent strains in the region were 176, 001/072 and 014/020, which is consistent with the present study [4]. The same distribution of the dominant *C. difficile* strains (027, 001/072 and 014/020) was found in the EUCLID study performed in Europe during 2012 and 2013, although the rate of 027 ribotype was lower than the one obtained in the present study [15]. In the last published study addressing *C. difficile* ribotypes Europe-wide, ribotype 027 remained the most prevalent, at the mean prevalence of 11.5% across 5 years, followed by ribotypes 001, 078, 014 and 020 [16]. On the contrary, in a recent study performed in North Macedonia, around 48% of isolates belonged to 001/072 [17]. As opposed to the ribotypes widely distributed across Europe, in a study performed in 2012–2013 including 482 European hospitals, some ribotypes had evidence of clear within-country clustering: ribotype 356, found only in Italy; ribotype 018, predominantly isolated in Italy; and ribotype 176, with distinct Czech and German clades [18].

An important observation of the present study is that, although ribotype 027 remained the most prevalent strain, a significant decrease in its rate was noted during the 3-year period. Compared to 95.6% in 2014/2015, this strain was isolated in 52.6% of the cases in 2017/2018, which was accompanied by the decrease in the overall number of patients treated for CDI.

In 2009, authors from Netherlands were the first to observe a similar, declining trend in the prevalence of ribotype 027, due to a responsible use of antibiotics and other preventive measures [19]. Although international comparisons are difficult, a decline in the rate of CDI caused by 027 ribotype was also reported by other countries, such as Belgium and Great Britain [20–22]. As opposed to these results, Italy and Germany reported the increase in the rate of patients with ribotype 027 infection reaching 38% and 30%, respectively [23, 24]. In Cyprus, its prevalence increased each year, from 28% in 2011 to 89% in 2015 [16].

The decline in the prevalence of the ribotype 027 infection, and CDI in general in Great Britain was the result of the surveillance over antibiotic usage, mainly fluoroquinolones [22]. Namely, one of the explanations for the spread of this ribotype in the epidemic era is fluoroquinolones overuse, considering that NAP1/BI/027 strain is resistant to these antibiotics. In the present study, the patients infected with ribotype 027 were more often pretreated with fluoroquinolones comparing to other patients. Furthermore, the study demonstrated a significantly lower rate of the patients treated with fluoroquinolones during the second study period. It might be one of the explanations for the decrease in the rate of ribotype 027 infection and the overall number of CDI patients in 2017/2018.

Ribotype 027 caused a severe form of CDI more often than other ribotypes, but it did not affect mortality and recurrence rates. There are controversial reports concerning the influence of the ribotype 027 on the CDI severity, recurrence and mortality rates. It was assumed that patients infected with NAP1/BI/027 strain develop more severe CDI forms and have a greater risk of experiencing recurrence, complications, and death [25–28]. The Canada-wide CDI study analyzed the role of strain type and patient age on the severity of CDI: a severe outcome, defined as CDI requiring intensive care unit admission, colectomy, or death within 30 days after diagnosis, was detected in 12.5% of patients with ribotype 027 and 5.9% of patients with other ribotypes [28]. However, the epidemiology of *C. difficile* is changing rapidly, and a number of studies suggest that strain type, including NAP1/BI/027, is not associated with the severity of the disease [29, 30].

In the study reported by Portuguese authors, the risk factors for fatal outcome were older age, leukocytosis, renal failure, and fatal comorbidities according to McCabe score [31]. The results of the present study also indicate that, in the subgroup of patients infected with ribotype 027, higher case fatality ratio was associated with leukocytosis, renal failure and higher CRP.

According to Michel and Gardner, the 30-days all-cause mortality in patients with CDI varied from 9 to 38%, and in-hospital mortality ranged from 8 to 37.2% [32]. In-hospital mortality in the present study was 18.8% and 18.2% in patients suffering from 027 and non-027 ribotype infection, respectively.

This study addresses a current disease, which in the past two decades presented not only a notable problem mainly for hospitalized patients but also a burden for healthcare systems worldwide. To the best of our knowledge, this is the only study regarding the distribution of the *C. difficile* ribotypes in Serbia after 2015 and after the registered peak in the number of CDI patients. Its prospective character and the comparison of the ribotype prevalence during two intervals 3 years apart are the main advantages of the present study.

The study has potential limitations. The major limitation is the small sample size, suggesting that the obtained results must be carefully interpreted, primarily those addressing the association between the ribotype 027 and the disease severity, death and recurrence rates. In addition, the number of strains, only 19, obtained during the second study period is particularly small. Nevertheless, the authors consider that the declining trend of the ribotype 027 infection rate and its association with a lower fluoroquinolone usage is beyond doubt.

## Conclusions

Ribotype 027 is the most prevalent ribotype among CDI patients in Serbia, with a declining rate over a 3-year period. It should be emphasized again that these findings must be taken with caution, since they were obtained from the analysis of only 64 patients. Ribotype 027 infection was associated with prior fluoroquinolone treatment and a severe form of the disease, but it did not affect death and recurrence rates. Further studies involving more patients need to be conducted in order to confirm these results or establish new trends in the distribution of the *C. difficile* ribotypes in Serbia.

## Data Availability

The data that support the findings of this study are available on request from the corresponding author [I.M.]. The data are not publicly available since containing information compromise research participant privacy/consent.
